# Fungal Keratitis Caused by *Drechslera* spp. Treated with Voriconazole: A Case Report

**DOI:** 10.1155/2013/626704

**Published:** 2013-09-18

**Authors:** Margarita I. Echavez, Archimedes Lee D. Agahan, Noel S. Carino

**Affiliations:** External Disease and Cornea Service, Department of Ophthalmology and Visual Sciences, Sentro Oftalmologico Jose Rizal, Philippine General Hospital, University of the Philippines Manila, Taft Avenue, Ermita, 1000 Manila, Philippines

## Abstract

*Objective*. To present a case of *Drechslera* spp. keratitis treated with topical Voriconazole. *Method*. A case report. *Results*. A 52-year-old diabetic male presented with a one-week history of foreign body sensation of the left eye, self-medicated with Neomycin, Polymyxin B, and Dexamethasone eye drops, and was diagnosed to have bacterial conjunctivitis, which was treated with Levofloxacin drops. The patient developed a corneal opacity after 2 days and was initially seen with a visual acuity of counting fingers on the left eye, with a 3 mm central corneal ulcer with feathery borders. No hypopyon was noted. The right eye had a visual acuity of 20/20 and had unremarkable findings. Corneal scraping of the ulcer showed no organisms on Gram and Giemsa stain. Cultures were positive for *Drechslera* spp. and patient was started on Natamycin drops every 15 minutes, Atropine drops 3× a day, and Levofloxacin was continued every 4 hours. The ulcer increased to 4 mm, the infiltrates became deeper involving the midstroma, and there was appearance of a 2 mm hypopyon. Natamycin was shifted to Voriconazole eye drops every 15 minutes. There was note of a decrease in the size of the ulcer and clearing of the infiltrates with the new treatment regimen. Final visual acuity after 29 days of treatment was 20/40 with note of a slight corneal haze in the area of the previous ulcer. *Conclusion*. Voriconazole may be safe and effective in the treatment of *Drechslera* keratitis. There was no perforation and there was immediate decrease in the size of the ulcer. This is the first known case of *Drechslera* keratitis treated with Voriconazole eye drops in the Philippines.

## 1. Introduction

Central microbial keratitis is one of the major causes of ophthalmic morbidity and visual loss. Worldwide, the reported incidence of fungal keratitis is 17% to 36%, caused by fungi, most commonly *Fusarium* and *Aspergillus* and other less common species like *Candida*, *Curvularia*, and *Monosporidium*, among others [1]. Organisms belonging to *Drechslera* species are rare causes of human infection and are considered opportunistic pathogens; however, it has been seen in healthy hosts [2]. Only 10 cases of human infection with these organisms have been reported in the literature [3]. We report the first case of fungal keratitis due to *Drechslera *spp. treated with topical voriconazole. 

## 2. Case Report

**Figure 1:**
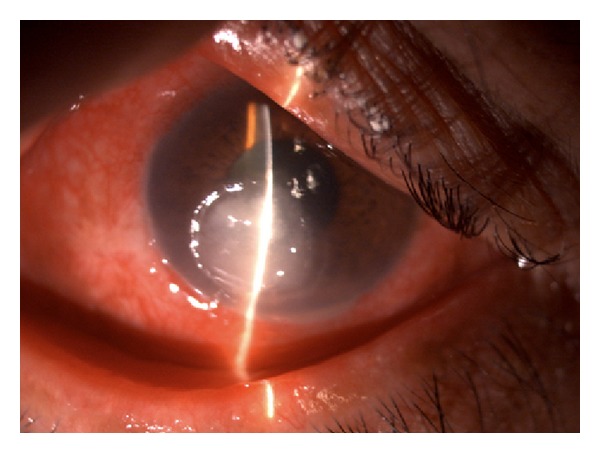
Day 11 since initial examination.

**Figure 2:**
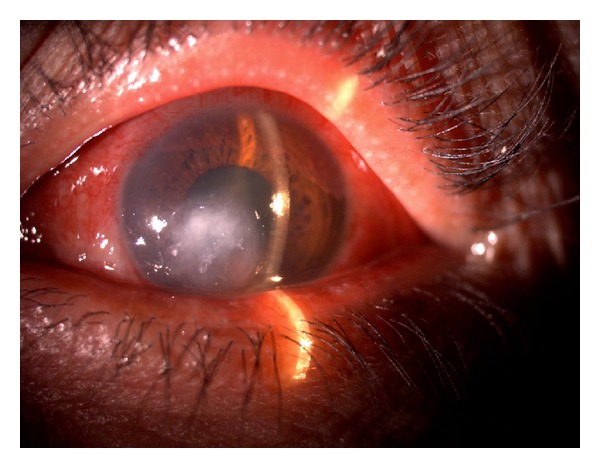
Day 12 since initial examination (Day 1 on Voriconazole).

**Figure 3:**
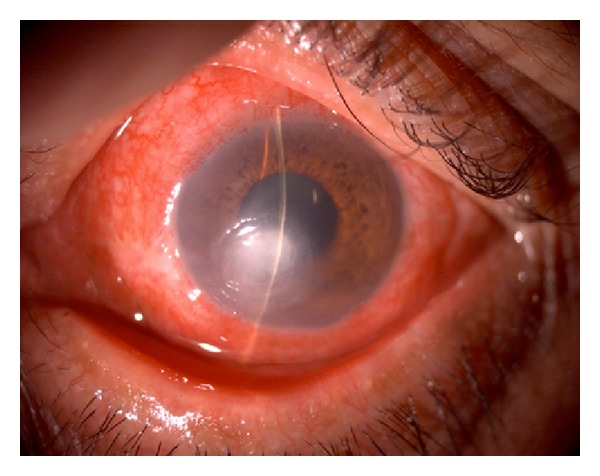
Day 14 since initial examination (Day 3 on Voriconazole).

**Figure 4:**
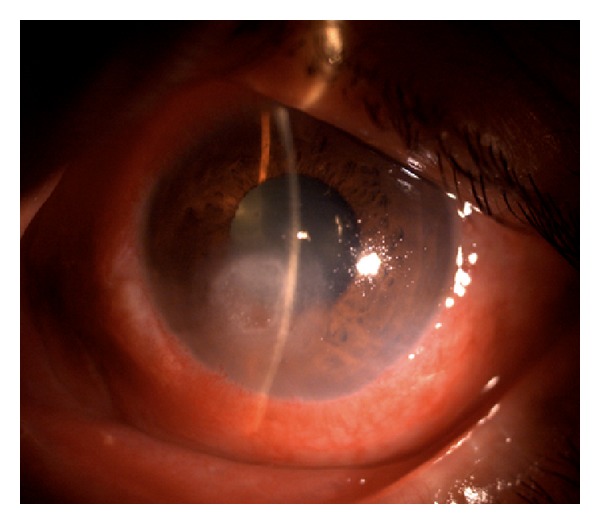
Day 16 since initial examination (Day 5 on Voriconazole).

**Figure 5:**
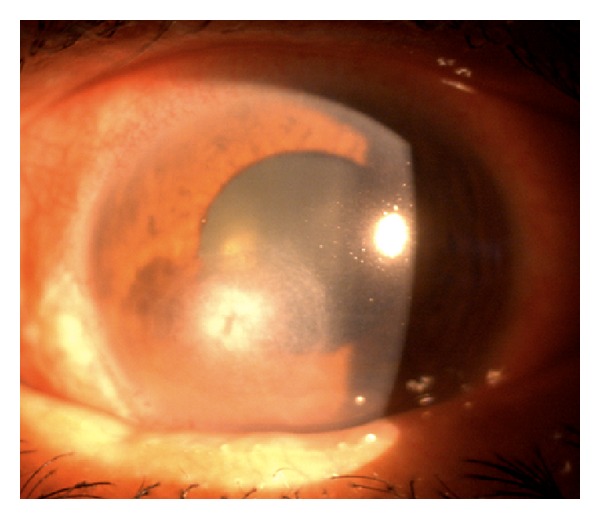
Day 22 since initial examination (Day 11 on Voriconazole).

**Figure 6:**
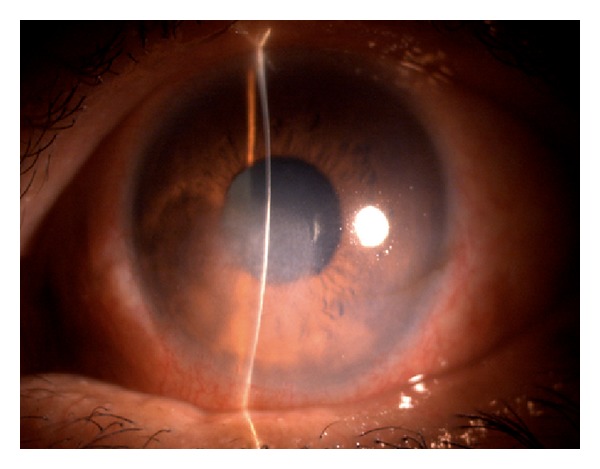
Day 29 since initial examination (Day 18 on Voriconazole).

A 52-year-old diabetic male presented with a one-week history of foreign body sensation of the left eye. Patient self-medicated with Neomycin, Polymyxin B, and Dexamethasone eye drops. He consulted a private eye doctor and was diagnosed to have bacterial conjunctivitis and was started on Levofloxacin drops. However, patient developed a corneal opacity after 2 days and consulted our institution. He was initially seen with a visual acuity of counting fingers on the left eye, with a 3 mm central corneal ulcer with feathery borders. No hypopyon was noted. The right eye had a visual acuity of 20/20 and had unremarkable findings. Corneal scraping of the ulcer was performed which showed no organisms on Gram and Giemsa stain. Cultures were positive for *Drechslera* spp. and patient was started on Natamycin drops every 15 minutes, Atropine 3× a day, and Levofloxacin was continued every 4 hours. The ulcer increased to 4 mm, the infiltrates became deeper involving midstroma, and there was an appearance of a 2 mm hypopyon (Figure 1). Natamycin was shifted to 10 mg/mL Voriconazole eye drops every 15 minutes and Levofloxacin q4 hours and Atropine drops 3× a day were continued. There was note of a decrease in the size of the ulcer and clearing of the infiltrates with the new treatment regimen (Figure 2). There was gradual decrease of the infiltrates and opacity (Figures 3, 4, and 5). Final visual acuity after 29 days of treatment was 20/40 with note of a slight corneal haze in the area of the previous ulcer (Figure 6). 

## 3. Discussion

A presumptive diagnosis of fungal keratitis requires empirical therapy. Current treatment and management of such cases include superficial keratectomy and topical antifungal medications. Natamycin is the drug of choice for filamentous fungal infection while Fluconazole is recommended for *Candida* infection. Amphotericin B is recommended for yeast infection and may be required for nonresponding cases. Other medications have been tried with moderate success. The infection typically takes a long time to heal and corneal perforation may occur in patients with untreated or partially treated keratitis which may require surgical intervention [1]. In the case presented, *Drechslera* spp. was cultured from the corneal ulceration scraping. This did not respond to the initial treatment of Natamycin; hence, medication was shifted to Voriconazole. A study by Sheridan [2], however, showed that a case of *Drechslera* keratitis did respond to Natamycin and visual acuity improved from 20/400 to 20/40, but this was not the case in our patient. It is, therefore, important to do close followup of these cases and to do trial of medications that may be more effective in treating the disease.

## 4. Conclusion

Voriconazole may be safe and effective in the treatment of *Drechslera* keratitis. There was no perforation, and there was immediate decrease in the size of the ulcer. This is the first known case of *Drechslera* keratitis treated with Voriconazole eye drops in the Philippines.

## References

[1] Mravicic I, Dekaris I, Gabric N An overview of fungal keratitis and case report on trichophyton keratitis. *Collegium Antropologicum*.

[2] Sheridan JE (1976). *Drechslera* spp. and other seed-borne pathogenic fungi in New Zealand cereals. *New Zealand Journal of Agricultural Research*.

[3] Rolston KV, Hopfer RL, Larson DL (1985). Infections caused by *Drechslera* species: case report and review of the literature. *Reviews of Infectious Diseases*.

